# The TECH@HOME study, a technological intervention to reduce caregiver burden for informal caregivers of people with dementia: study protocol for a randomized controlled trial

**DOI:** 10.1186/s13063-017-1796-8

**Published:** 2017-02-09

**Authors:** Agneta Malmgren Fänge, Steven M. Schmidt, Maria H. Nilsson, Gunilla Carlsson, Anna Liwander, Caroline Dahlgren Bergström, Paolo Olivetti, Per Johansson, Carlos Chiatti, on behalf of the TECH@HOME Research Group

**Affiliations:** 1 Department of Health Sciences, Health Sciences Centre (HSC), Lund University, Baravägen 3, 222 41 Lund, Sweden; 2 Memory Clinic, Skåne University Hospital, Malmö, Sweden; 3 Scientific Direction, Italian National Institute of Health and Science on Ageing (INRCA), Ancona, Italy; 4 Institute of Medicine, The Sahlgrenska Academy University of Gothenburg Department of Internal Medicine (Endocrinology), Gothenburg, Sweden

**Keywords:** Cognitive impairment, Informal care, Caregiver burden, Health-economic evaluation

## Abstract

**Background:**

It is estimated that global dementia rates will more than triple by 2050 and result in a staggering economic burden on families and societies. Dementia carries significant physical, psychological and social challenges for individuals and caregivers. Informal caregiving is common and increasing as more people with dementia are being cared for at home instead of in nursing homes. Caregiver burden is associated with lower perceived health, lower social coherence, and increased risk of morbidity and mortality. The aim of this trial is to evaluate the effects of information and communication technology (ICT) on caregiver burden among informal caregivers of people with dementia by reducing the need for supervision.

**Methods/design:**

This randomized controlled trial aims to recruit 320 dyads composed of people with dementia living in community settings and their primary informal caregivers. In the intervention group, people with dementia will have a home monitoring kit installed in their home while dyads in the control group will receive usual care. The ICT kit includes home-leaving sensors, smoke and water leak sensors, bed sensors, and automatic lights that monitor the individual’s behavior. Alerts (text message and/or phone call) will be sent to the caregiver if anything unusual occurs. All study dyads will receive three home visits by project administrators who have received project-specific training in order to harmonize data collection. Home visits will take place at enrollment and 3 and 12 months following installation of the ICT kit. At every home visit, a standardized questionnaire will be administered to all dyads to assess their health, quality of life and resource utilization. The primary outcome of this trial is the amount of informal care support provided by primary informal caregivers to people with dementia.

**Discussion:**

This is the first randomized controlled trial exploring the implementation of ICT for people with dementia in a large sample in Sweden and one of the first at the international level. Results hold the potential to inform regional and national policy-makers in Sweden and beyond about the cost-effectiveness of ICT and its impact on caregiver burden.

**Trial Registration:**

ClinicalTrials.gov, NCT02733939. Registered on 10 March 2016.

**Electronic supplementary material:**

The online version of this article (doi:10.1186/s13063-017-1796-8) contains supplementary material, which is available to authorized users.

## Background

Worldwide, 47.5 million people are living with dementia, and it is estimated that their number will double by 2030 and more than triple by 2050 as a result of the world’s aging population [1]. Dementia has a dramatic impact on individuals but also places a staggering burden on families and societies, which makes it a public health priority [2]. In Sweden, there are approximately 160,000 people who are diagnosed with some form of dementia. During the last 15 years, the actual number of people with a dementia diagnosis has increased, and approximately 25,000 new dementia cases are diagnosed every year in Sweden [3] with Alzheimer’s disease being the most common diagnosis. The majority of people with dementia live in their own homes despite considerable disability. Thus, the costs to municipalities for home care services, housing adaptations and technical devices have increased [4].

Informal caregiving, provided by relatives or friends, has increased along with the prevalence of the disease and more people with dementia are being cared for at home instead of in nursing homes. A study by Nordberg and colleagues [5] suggested that the amount of informal care that people with dementia receive is greater than the amount of formal care (6 h/day compared to 1 h/day) even in the generous Scandinavian welfare systems. In other European countries, such as Italy, the number of hours of care provided by primary informal caregivers has been estimated to be as high as 50 h per week, which includes direct care provision and supervision activities [6]. There are different methods to evaluate the economic impact of the time spent in care activities by informal caregivers, and this leads to a clear heterogeneity of research findings [7]. However, all available figures suggest a significantly high societal burden. In Sweden, which has fewer than 9 million inhabitants, the National Board of Health and Welfare estimated that the annual cost of dementia was about SEK62.9 billion (USD7.34 billion) or approximately SEK400,000 (USD46,660) per person with dementia, of which the societal cost reached approximately SEK10 billion (USD1.17 billion) [4]. The size of such burden and its likely increase over the next few years urges policy-makers and professionals to tackle this phenomenon with new and innovative measures to ensure the sustainability of the existing welfare states [1].

While a cure for dementia is not available yet, an area of interest for professionals and policy-makers is support for caregivers. As a consequence of their intensive and lengthy engagement (a person with dementia can live up to 20 years after diagnosis) [8], dementia caregivers are at risk of experiencing anxiety and stress, which leads to a higher rate of mortality compared to their noncaregiver counterparts [9]. Informal caregivers with high caregiver burden report lower perceived health and higher care services and drug utilization compared to caregivers with lower burden [10]. Interventions that support caregivers of people with dementia do not only hold the potential to reduce the burden but could also lead to beneficial effects for the people with dementia, e.g., delaying nursing home placements and reducing the use of antipsychotic medications [11]. Several innovative strategies have been tested to reduce the burden of care: providing practical assistance, education [12], role-training interventions [13], family intervention programs [14], and psychoeducational interventions [15]. Results of these approaches are inconsistent, but it has been suggested that multicomponent interventions that combine different strategies have the greatest impact on reducing care burden [12, 16, 17].

In recent years, multicomponent interventions that include a combination of innovative technologies and more “traditional” care services have become increasingly popular in the field of dementia care. As caregivers’ supervision time, necessary to prevent dangerous and harmful events for the person with dementia, constitutes the largest share of informal caregiving, new technologies for environmental safety and control can potentially reduce the time needed for supervision. However, studies in this area have rarely been well-designed epidemiological studies, but they have rather tended to be stand-alone pilots, qualitative studies or uncontrolled trials based on convenience samples, generally conducted by researchers in the technology field [18].

The UP-TECH project was a recent study about the impact of new technologies for dementia caregivers, involving care staff, engineers, epidemiologists and health economists, which developed and tested a prototypical ICT solution combined with a case-management intervention in more than 100 households in Italy [19]. UP-TECH used home monitoring kits to monitor adverse and dangerous events (e.g., smoke, water leaks, unexpected opening of doors or windows, prolonged absence from bed) through a wireless network and a control unit that sent alarms to the caregivers via mobile phone [20]. Preliminary results of the UP-TECH study have been promising, and user satisfaction with the solution was high [21]. Another relevant study is the ATTILA trial in England [22]. ATTILA uses assistive technology and telecare (ATT) to support independent living at home for people with dementia. The hypothesis of the study is that people with dementia who receive ATT will be less likely to go into institutional care than those who receive equivalent community services without ATT. Results for this study are not yet available as the study is still ongoing.

The TECH@HOME study builds on the experiences of the UP-TECH study and will test the impact of a newly designed technology to support caregivers of people with dementia who are still living in the community, in terms of effectiveness and cost-effectiveness. We hypothesize that technologies can substitute for some of the time caregivers spend in supervision and monitoring activities for people with dementia. The decreased time is expected to reduce the caregiver burden, and thus result in an overall improvement of quality of life for carers and people with dementia.

## Methods/design

### Study design

**Fig. 1:**
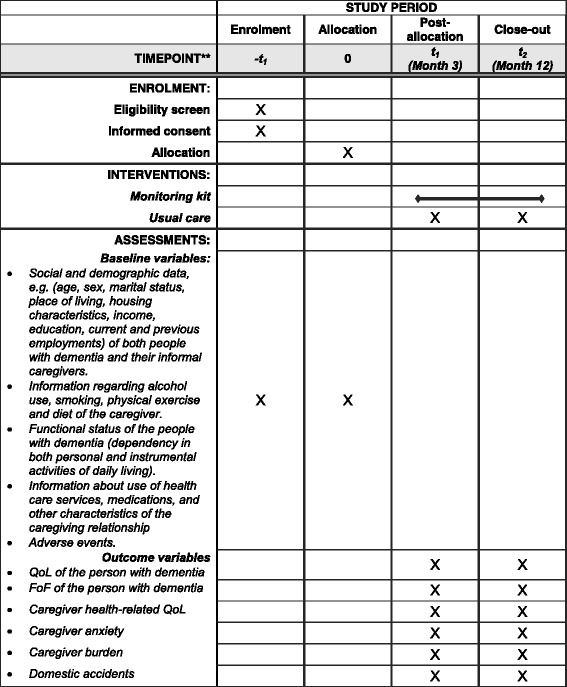
Standard Protocol Items: Recommendations for Interventional Trials (SPIRIT) figure of TECH@HOME

TECH@HOME is a randomized controlled trial to test the effectiveness of an innovative technological intervention among people with dementia and their primary informal caregivers in Sweden. The intervention will last 12 months. A total of 320 dyads, including people with dementia and their primary informal caregivers (640 participants in total), will be recruited. These dyads will be randomized either into an intervention or a control group (see Fig. 1 for the Standard Protocol Items: Recommendations for Interventional Trials (SPIRIT) figure and for the complete SPIRIT Checklist see Additional file 1). All dyads, both the intervention and control groups, will receive three home visits (at baseline and 3 and 12 months after enrollment) from a dementia nurse in charge of data collection.

In the intervention group, besides usual care, the people with dementia will have a technological home monitoring kit installed in their home (as described under the “Intervention tested” section) while dyads in the control group will only receive usual care. Usual care for people with dementia in Southern Sweden can vary. In the target area, people with dementia usually receive comparable pharmaceutical treatment depending on the type of dementia, as prescribed by a general practitioner or a specialist at a memory clinic. Patients of memory clinics receive regular follow-ups depending on the type of diagnosis and on-going treatment response, while the majority of people with dementia are treated and monitored at a primary health care center and by the municipal health care services. Social workers from the municipality (“Biståndshandläggaren”) where the person resides, together with district nurses, have a meaningful role in tailoring the care plan by mediating access to other care services such as respite care homes, home help, and (dementia) nurse home visits. Use of such services depends on the specific needs of the person with dementia, which can also be unrelated to dementia, but rather dependent upon concomitant health and social issues.

### Intervention tested

**Fig. 2:**
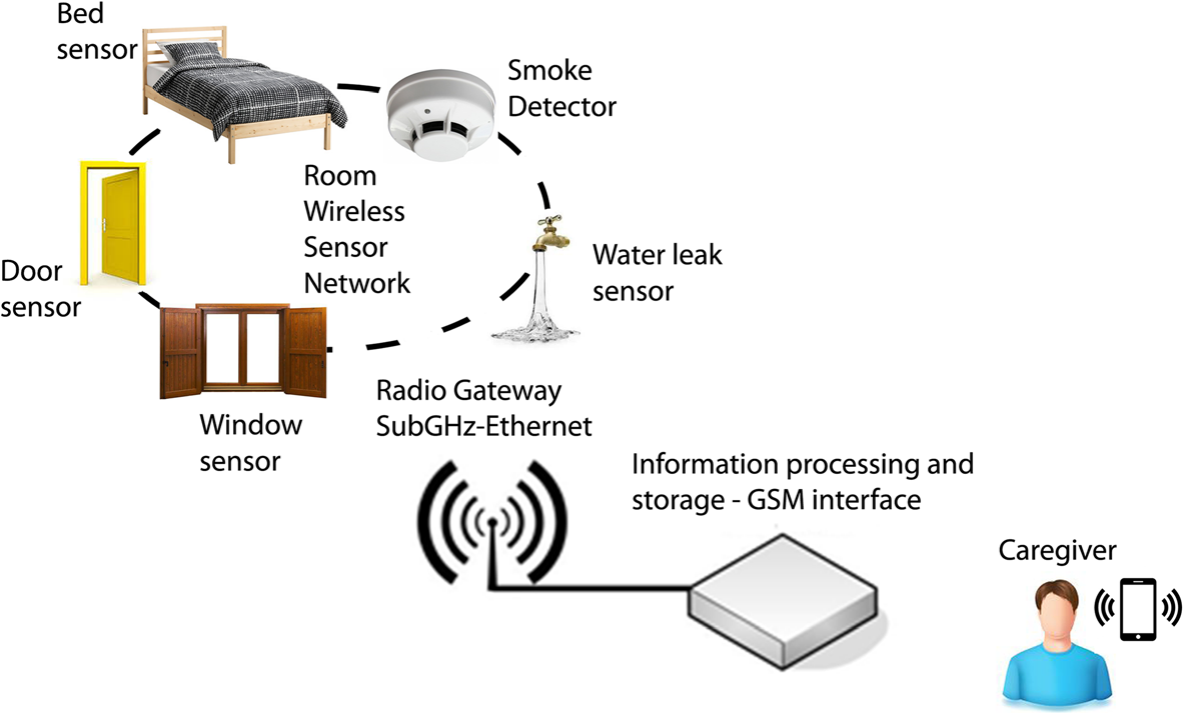
Concept of the monitoring kit used in the project

The intervention includes the installation and use of a technical monitoring kit in the home for 12 months. The home monitoring kit includes devices that are easy to use and do not require significant technical expertise for installation and maintenance. The kits will be composed of a control unit and a set of sensors that immediately notify caregivers, through their phones, of any potential risks for the person with dementia. The kit will have home-leaving sensors, bed-occupancy sensors, smoke and water leak sensors, automatic lights, and other interactive functions. These devices will be connected to a single-board microcontroller that will transmit alarm messages to the caregivers in case of need. The monitoring kits will be assembled by an external contractor and installed by a handyman who will also train the caregivers about their use (see Fig. 2). The handyman will be trained and receive a manual on how to provide a standardized introduction to all caregivers, who will be probed to ask for additional information. Written information for users will be developed in collaboration with caregivers, followed by a subsequent field test.

#### Functionality of the kit

In case of potentially harmful or dangerous situations, the control unit will notify the selected phone number with a text message and/or phone call. If the caregiver does not answer, the control unit will contact alternate numbers in the order specified (maximum six home and mobile phone numbers chosen by the caregivers). The home monitoring kit will notify the caregivers about potentially dangerous situations such as when the person with dementia:

- leaves the home without notice, since this might occur in a moment of disorientation and the person might wander away and get lost (magnetic contacts must be placed on critical doors and windows)
- leaves a water tap open and floods the room (water sensors must be placed in critical places)
- forgets something on the stove while cooking and causes a fire (smoke detection sensors must be placed in critical places)
- gets out of bed during the night and does not come back within a specified time interval (bed-occupancy sensor and configurable time control)
- never goes to the bathroom in 24 h (passive infrared sensor and configurable time control)
- never opens refrigerator door in 24 h (magnetic contact and configurable time control)

Depending on the personal choice of the person with dementia and/or their caregiver, the kit will also:

- automatically turn on a light to help avoid a fall (via a bed-occupancy sensor, passive infrared sensors and automatic lights) when the person gets out of bed during the night
- alert the caregiver when the temperature in the house falls or rises excessively (temperature sensor with configurable threshold)
- act as burglar alarm in some areas of the home (via magnetic contacts and passive infrared sensors)

#### Installation and usability requirements

The installation of the home monitoring kit will be simple and quick because the dyads participating in the intervention are particularly vulnerable. A checklist will be created for the handymen performing the installation, thus reducing the variability of their intervention in the homes. Wireless communications and battery power will be used for all of the sensors in the kit. The installation of the kit will not be invasive as people with dementia could get confused by the sensors. Components will be hidden whenever possible in accordance with the choices of the study participants (both people with dementia and their caregivers). The home monitoring kits will also be easy to use, so the dyads will only be trained to perform simple procedures such as activating the device. In order to achieve this, only a few buttons will be available on the control unit with simple LED lights to indicate the state of the control functions and the correct running state of the home monitoring kit. Activation and deactivation of some functions will also be available through text messaging. A phone number will be available for the people with dementia and their caregivers in case they need technical assistance with the equipment after the home installation. Technicians will be able to provide assistance remotely in most cases.

#### Security requirements

One of the study goals is to assess the impact on care burden and anxiety among informal caregivers through the use of technology. In order to do this, false alarms will be avoided as much as possible for the same reasons. In the case of potentially dangerous situations, such as a fire or water leakage, the system will emit an acoustic alarm in accordance with the choices of the person with dementia and their family. Any other type of acoustic signal will be avoided so as not to frighten the users or cause confusion.

### Primary and secondary outcomes

The primary outcome of the study is the amount of informal care support provided to the individual with dementia by the primary informal caregiver.

Secondary outcomes of the study include:

1. Quality of life of the person with dementia
2. Fear of falling of the person with dementia
3. Caregiver health-related quality of life
4. Caregiver anxiety
5. Caregiver burden
6. The incidence of domestic accidents
7. The cost-effectiveness of the technological intervention

### Eligibility criteria

People with dementia living in the area of Region Skåne (Southern Sweden), together with their primary informal caregiver will be invited to participate in the study.

The inclusion criteria for the study that are applied to the person with dementia are:

- a diagnosis of major neurocognitive disorders with mild to moderate severity (*Diagnostic and Statistical Manual of Mental Disorders, version 5* (DSM-5)) following the new diagnostic criteria of the American Psychiatric Association [23]
- a score of between 14 and 24 on the Mini Mental State Examination (MMSE-SR)
- a score of between 1 and 5 on the Global Deterioration Scale (GDS) [24]
- community dwelling
- able to speak and understand Swedish
- have at least one informal caregiver

Those not meeting the inclusion criteria will not be eligible for the trial. In addition, specific exclusion criteria will be:

- lack of informed consent
- being fully dependent on caregiver support for the activities of daily living (ADL)
- presence of severe diseases associated with a life expectancy of less than 6 months
- intention of moving to institutionalized care during the study period
- unwillingness to use technological devices for home assistance and safety
- being enrolled in another ongoing trial
- substance use disorder (DSM-5)

Informal caregivers are defined as those people who provide care and support to an individual with dementia on a regular basis without any form of financial compensation. Exclusion criteria applied to the informal caregiver are:

- lack of informed consent
- unwillingness to use technological devices for home assistance and safety
- presence of severe diseases associated with a life expectancy of less than 6 months
- being already enrolled in another ongoing trial

The person with dementia and their caregiver can either be cohabitating or live in different homes.

Informed consent will be actively sought from both parties. Participation in the study is completely voluntary and participants will receive verbal and written information about the study prior to their participation and during the study implementation.

### Screening and recruitment

Participants will be recruited at the Memory Clinic at Ängelholm’s Hospital in Sweden, which is coordinated by PJ. The neurologists and nurses working at the Memory Clinic will review their list of patients to identify those meeting the inclusion criteria and send an invitation letter to participate that also describes the aims and characteristics of the study. We estimated a participation rate of 50% based on previous studies in this area [25]. In addition, we plan to distribute a brochure describing the characteristics of the project among the primary health care centers in the surrounding areas, informing potential candidates about the study. The potential participants (both people with dementia and their caregivers) will then be assessed by the dementia nurse coordinating the field activities (CDB) to double-check whether the inclusion criteria are met and to further assess the exclusion criteria. Eligible dyads will then be asked to sign the informed consent for the study. Basic information (age gender and town of residence) about those participating in the study will be compared with that of the people meeting the inclusion criteria who are enlisted in the Memory Clinic database, only on aggregate level, for the purpose to assess potential participation bias.

### Randomization and allocation

Following written informed consent, names of the dyads enrolled will be sent directly to the principal investigator of the study (CC) who will perform the randomization. Dyads will be randomized (according to a 1:1 ratio) either to the experimental arm (receiving the technological intervention), or to the control arm (receiving usual care). The allocation sequence will be generated using the statistical software STATA (StataCorp, College Station, TX, USA).

### Assessments and procedures for data collection

All participants signing the informed consent to participate will be enrolled in the study and will receive three home visits by a dementia nurse trained in the methodology used in the project. Informed consent will be actively sought both from people with dementia and their caregivers. In case of people declared legally incompetent, informed consent will be requested from a family member or from another person legally appointed by a judge to act as substitute. Home visits will occur at enrollment and after 3 and 12 months. Before the home visit, the self-administered sections of the questionnaires will be mailed to the caregivers participating in the study. The home visits will take approximately 1 h, during which the remaining sections of the questionnaire will be administered to the study participants and a general check of the self-administered forms will be made by the dementia nurse. Those in the experimental arm will receive the installation of the home monitoring kit at latest 1 month following the baseline home visit.

A 12-section study-specific questionnaire has been designed. The questionnaire sections addressing the people with dementia will be administered by the dementia nurse and cover the following domains: (A) sociodemographic information, (B) cognitive function, (C) ADL, (D) health and quality of life, and (E) use of health care resources.

The sections addressing the informal caregiver will be self-administered and cover the following domains: (F) sociodemographic information, (G) lifestyle, (H) health and quality of life, (I) mental health and wellbeing, (J) caregiver burden, (K) caregiving time, and (L) use of health care resources.

### Primary outcome measurement

The amount of informal care provided by caregivers to the people with dementia, considered as a proxy of the caregiver burden, will be measured in h/week and assessed using a specific section of the Resource Utilization in Dementia (RUD) instrument [26]. The change from baseline to months 3 and 12 will be considered as primary outcome for the analysis.

### Secondary outcome measurement

Quality of life of the person with dementia will be measured using the Quality Of Life in Alzheimer’s Disease (QOL-AD) instrument [27], which has been developed in collaboration with caretakers, caregivers and experts in dementia care to ensure validity. Previous studies suggest that the instrument’s validity and reliability are satisfactory in a study population of people with dementia: Cronbach’s *α* ranges from 0.84 to 0.88 for people with dementia and caregivers and 1-week test-retest reliability is also acceptable (intraclass correlation coefficient (ICC) = 0.76 for people with dementia and 0.92 for caregivers) [28].

Fear of falling of the person with dementia will be measured using the 16-item Falls Efficacy Scale-International (FES-I) [29–31]. The evaluation of the Swedish version of the instrument showed high internal reliability (Cronbach’s *α* = 0.95) and an interitem correlation averaging 0.55 [32]. Quality of life of the caregiver will be assessed using the EuroQol, 5 dimensions, 3 levels (EQ-5D-3 L) health survey. This standardized instrument to measure health outcomes [33] has been used and validated in previous Swedish studies [34, 35], showing both a good internal reliability (Cronbach’s *α* = 0.73) and validity [36]. Caregiver anxiety will be assessed using the anxiety component of the Hospital Anxiety and Depression Scale (HADS) [37]. HADS is a 14-item scale; seven of the items relate to anxiety and seven relate to depression; each item is a Likert scale rated from 0 to 3, and this means that the overall score for either anxiety or depression will range from 0 to 21. Cronbach’s *α* for HADS-Anxiety varies from 0.68 to 0.93 (mean 0.83) and for HADS-Depression from 0.67 to 0.90 (mean 0.82) [38]. The Zarit Burden Inventory (ZBI) will be used to measure the level of caregiver burden [38]. This is the instrument most consistently used in dementia caregiving research [39], and it is often used to measure the change of caregiver burden over time, resulting from the progression of the disease severity of the care recipient or from interventions aimed at reducing burden. The revised version of the ZBI with 22 items will be used, as this has shown both a high reliability (Cronbach’s *α* from 0.88 to 0.91) and validity (it is highly correlated with a single global burden rating, *r* = 0.71) [40]. Domestic accidents are defined as accidental falls, bruises, cuts, episodes of wandering outside the home, burns and fires, flooding, or other events which potentially or concretely harm the people with dementia. The incidence of domestic accidents will be assessed using an ad-hoc form.

### Other measurements and adverse events

Basic social and demographic data, such as age, sex, marital status, place of living, housing characteristics, income, education and current and previous employments, will be collected from both the people with dementia and their informal caregivers. Information regarding alcohol use, smoking, physical exercise and diet of the caregiver will be retrieved using an ad-hoc module based on the forms of the Swedish National Health Survey.

The questionnaire developed for the study includes other instruments that have already been tested in studies addressing people with dementia. To assess the functional status of the people with dementia (dependency in both personal and instrumental activities of daily living (IADL)), the instruments of the interRAI suite will be used. As the full form of the RUD questionnaire will be administered, full information about use of health care services, medications, and other characteristics of the caregiving relationship will be available.

Although TECH@HOME is a nonpharmacological trial, the research team will actively monitor adverse events, i.e., any unfavorable and unintended sign, symptom, or disease temporally associated with the intervention, whether or not it is causally related to the study (e.g., falls, acute episodes of illness, etc.). This will mainly be done using two sources of information: (1) people with dementia and their caregivers will be instructed to refer to the clinical study coordinator in case of serious adverse events (requiring general practitioner support or hospital care) and (2) similarly, the clinical staff at the memory clinic will report in case any adverse event is acknowledged. Participants (both people with dementia and their caregivers) may be asked to withdraw if it is determined that participation in the trial does not represent the best interests of the participants themselves.

### Sample size calculation and statistics

According to Nordberg and colleagues [5], the mean number of hours spent in caregiving by informal caregivers of people with dementia ranges from 7.7 h per week (people with clinical dementia rating (CDR) 0.5) to 46.9 h per week (people with CDR 2.0). Wimo and colleagues [41] suggest that a standard deviation (SD) of 20 can be considered acceptable when using informal caregiving hours as an outcome for formal sample size calculation. Given these numbers, a sample size of 320 dyads randomized in a 1:1 ratio (160 per arm) is sufficient to detect a mean decrease in caregiving time of 1 h per day with a statistical power of 0.8 and a dropout rate of 20%, aiming at a final number of 128 dyads in the experimental arm and 128 dyads in the control arm.

The final database will be checked and cleaned using statistical routines after completion of each wave of assessments. All modifications made during this phase will be recorded in a specific file in order to allow for replicability of all operations made on the raw database.

All collected data will be made anonymous in accordance with the Swedish Data Protection Act (1998: 204) and coded in a database with access protection. The database will be stored on a server with continuous daily safety backup. Researchers will analyze data containing serial number encoded data without access to names or social security numbers. All information relating to the participants, the sampling framework, written consent, and the results from questionnaires are handled so that no unauthorized access to them can occur, and kept locked up. Only current researchers in the project will get access to the materials. Materials will be archived for 10 years.

The first step of the analysis will be exploratory in nature. A descriptive analysis of the sample will be conducted using univariate and bivariate statistical analyses with the aim of verifying the comparability of the study groups. Significant differences between exposures and outcomes will be compared using the chi-square test, Fisher’s exact test (in the case of categorical variables), the Mann-Whitney *U* test (for ordinal data such as the FES-I), the *t* test, or analysis of variance (ANOVA) test (for comparisons of continuous variables between groups according to a normal or non-normal distribution). Additionally, to check possible selection bias, the characteristics of the subjects in the sample will be compared, only on aggregate level, to those of the population with neurocognitive disorders included in the memory clinic register of the study area (e.g., age, sex, and place of living).

The primary outcome “time of informal care provided” will be compared between the two groups using analysis of covariance (ANCOVA). The analyses will be adjusted for variables which are nonhomogenously distributed in the two groups and controlling for other factors that the medical literature suggests are associated with the two outcomes (e.g., sex and age of caregivers, severity of dementia, and use of private care workers). The analyses of outcomes will be intention-to-treat (ITT).

### Economic evaluation

Among the secondary outcomes of the study, we will investigate the cost-effectiveness of the technological intervention, in case the overall intervention does not yield statistically significant improvements in the other primary and secondary study outcomes. Therefore, cost of illness, budget impact and cost-utility analyses will be performed using the perspective of the National Health Service [42] and in agreement with the ITT principle per the CHEERS statement [43].

1. Cost of illness analysis (COI) will define the value of the resources that are expended or foregone as a result of dementia. The COI will consider separately the perspective of the public health system, the users and the society in order to describe in a more comprehensive manner the real impact of the disease in the study context
2. Budget impact analysis (BIA) will measure the net cumulative effect of the technological intervention for dementia in the Swedish population. If the intervention can reduce the disease burden, it can also assure a reduction in terms of institutionalizations, number of hospitalization days, drug consumption, caregiver burden, etc.
3. Cost-utility analysis (CUA): defined as the ratio between the cost of the technological intervention and the benefit it produces in terms of the number of years lived in full health by the beneficiaries. The result of the analysis will be an incremental cost per quality-adjusted life year (QALY) gained.

Probabilistic sensitivity analyses (PSA) will also be performed on the health economics models using the software TreeAge Pro 2009. Results from the PSA will also be presented as an acceptability curve, graphically illustrating the probability of the intervention being cost effective over a range of willingness-to-pay values.

### Subgroup and secondary analyses

The implementation of a technological intervention can be considered “complex” according to the definition of the UK Medical Research Council [44]. This means that the study researchers need to identify and appraise all the intertwining components which are interacting and determining the outcomes themselves. Therefore, specific subanalyses are planned such as intervention integrity, dyad adherence to the study, and acceptance of the technology by the users. The research group plans to administer a further ad-hoc questionnaire at the end of the trial to both the people with dementia and the caregivers to assess their level of satisfaction with the technologies used. A specific ethical approval for this aspect of the study will be sought in the future.

### Handling of potential study bias

Risk of selection bias will be assessed by looking at the results of the enrollment process. Clinical staff will be instructed about how to explain, in very simple terms, what participation in the trial will entail. This is expected to reduce refusals among those people who are not very familiar with new technology. Considering the high level of “digital” education among the older population in Sweden, even compared to other Western countries, the likelihood of a selection bias should be minimized. The involvement of expert dementia nurses in the study also addresses the need to use research staff with a high level of know-how on how to relate with people with dementia and their families. This is also expected to reduce the dropouts and withdrawals during the study, thus increasing overall retention.

Other mediating and confounding variables might be associated with study outcomes, and these could lead to wrong/false results at the end of the trial. The use of a multidimensional questionnaire and the randomization process are expected to reduce such risks by providing several covariates that will be available for the statistical analyses to ensure the two study arms are comparable under several social, clinical and functional profiles.

Last but not least, the lack of adherence to the technology can constitute a major flaw for the study implementation and results. This risk will be minimized by using a standardized but thorough approach to the training of users (both people with dementia and their caregivers) with technologies, by using a very simple technological device, and by having the possibility to remotely monitor in real time the actual use of the devices. The study coordinator will be able to detect when a person is not using the technology and will promptly react in order to understand (and possibly address) the reasons behind the lack of use.

## Discussion

While the enormous technological progress made in recent years has put technologies, such as mobile devices and smart phones, in the reach of many, innovators in dementia care are just starting to explore the full potential of these developments to transform them into valuable products and services for users. There is indeed a lack of studies that evaluate the effectiveness and cost-effectiveness of these new technologically enriched interventions designed for people with dementia. Previous large-scale evaluations of the impact of telemedicine and telecare, such as the Whole Demonstrator System in the UK [45], did not include people with dementia despite Alzheimer’s disease being one of the most burdensome diseases in Europe [41].

This randomized controlled trial aims to evaluate the effects of new technologies on caregiver burden by reducing the time spent in supervision. The trial builds on previous promising results from the UP-TECH project in Italy [19]. The technologies used in TECH@HOME will include similar, but improved, home monitoring kits potentially leading to a greater impact on caregivers’ living and care conditions. In addition, while the UP-TECH study did not allow the researchers to estimate the impact of the technology as a “stand-alone” intervention (the monitoring kits were only given in combination with case-management support), TECH@HOME will overcome this limitation thanks to the possibility of comparing a group of technology users versus nonusers. Results from this intervention in dementia care in Sweden hold the potential to inform regional and national policy-makers in Sweden and beyond.

### Trial status

The trial started recruiting in March 2016 and the assessments are scheduled to continue until December 2017.

## Additional file

Additional file 1: Spirit Checklist. The additional file contains the populated SPIRIT Checklist of the TECH@HOME study. (DOC 120 kb)

## Abbreviations

ADL: Activities of daily living
ANCOVA: Analysis of covariance
ANOVA: Analysis of variance
ATT: Assistive technology and telecare
BIA: Budget impact analysis
CDR: Clinical dementia rating
CI: Confidence intervals
COI: Cost of illness analysis
CUA: Cost-utility analysis
HADS: Hospital Anxiety and Depression Scale
IADL: Instrumental activities of daily living
INRCA: Italian National Institute of Health and Science on Ageing
ITT: Intention-to-treat
MMSE-SR: Mini Mental State Examination
QALY: Quality-adjusted life year
RCT: Randomized controlled trial
RUD: Resource Utilization in Dementia
SD: Standard deviation
